# Supplementary material for: Inflammatory factors and risk of lung adenocarcinoma: a Mendelian randomization study mediated by blood metabolites
Source: Front Endocrinol (Lausanne). 2024 Aug 27;15:1446863. doi: 10.3389/fendo.2024.1446863 (PMC11384989; doi:10.3389/fendo.2024.1446863)
Supplement: Supplementary file 1 [file DataSheet1.zip › supplementary files/S2/GCST90200238/forest.pdf]

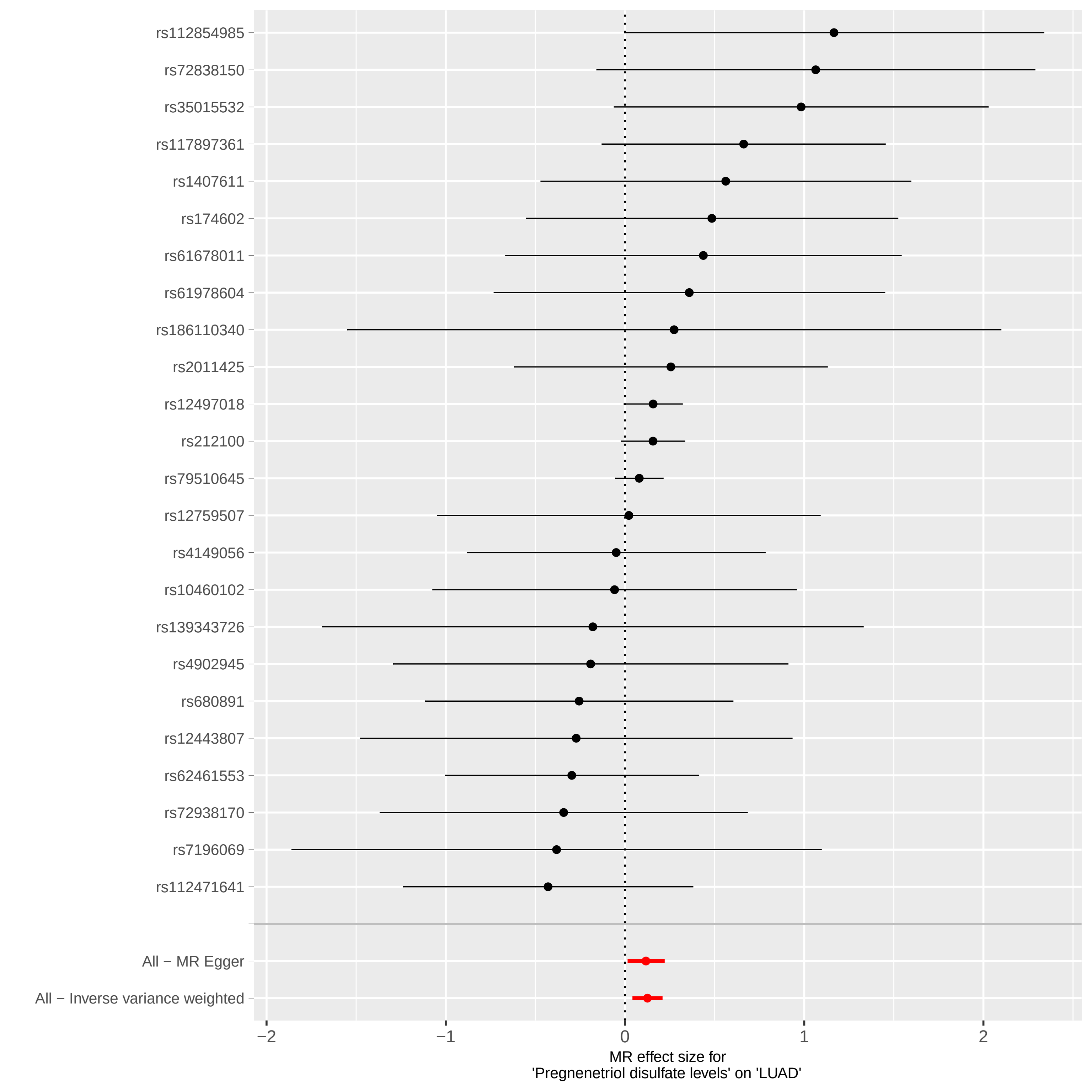

rs112854985
rs72838150
rs35015532
rs117897361
rs1407611
rs174602
rs61678011
rs61978604
rs186110340
rs2011425
rs12497018
rs212100
rs79510645
rs12759507
rs4149056
rs10460102
rs139343726
rs4902945
rs680891
rs12443807
rs62461553
rs72938170
rs7196069
rs112471641
All − MR Egger
All − Inverse variance weighted
−2
−1
0
1
2
MR effect size for
'Pregnenetriol disulfate levels' on 'LUAD'
